# The use of artificial intelligence and machine learning in the care of people with dementia: A literature review

**DOI:** 10.1192/j.eurpsy.2021.1144

**Published:** 2021-08-13

**Authors:** G. Belam, R. Nilforooshan

**Affiliations:** 1 Old Age Psychiatry, Unither House, Surrey and Borders NHS Foundation Trust, Chertsey, United Kingdom; 2 Abraham Cowley Unit, Surrey and Borders NHS Foundation Trust, Chertsey, United Kingdom

**Keywords:** Artificial Intelligence, machine learning, dementia, old age psychiatry

## Abstract

**Introduction:**

Artificial intelligence and machine learning are increasingly being researched within the field of psychiatry to find out what use it might be. With this review, therefore, we would like to assess what literature, if any, exists that answers the question of whether this technology can be useful for providing dementia care. We also wanted to consider the ethical questions of autonomy, consent and privacy when working with this vulnerable group of patients.

**Objectives:**

To identify and appriase the literature to assess the existing research landscape of the area of machine learning and AI, relating to the care of people with dementia.

**Methods:**

A literature search was conducted, searching the PsychInfo, Medline, PubMed and Embase databases. We assessed the quality of the research and considered what overall findings there were in the existing literature.

**Results:**

619 papers were identified, of which 28 related to the use of AI in the care of people with dementia. The papers were divided into categories to show the utility and effectiveness these technologies may have: 1: to alert caregivers to problems 2: to facilitate activities for people with dementia 3: to help plan care for people with dementia 4: to consider the ethical implications of the use of artificial intelligence and machine learning

**Conclusions:**

Despite a paucity of literature in the area, existing studies show potential, if used well, for technologies to be a useful addition to care of people with dementia. The experience of patients and their carers must be integral to their development and use.

